# The rise and fall of breakpoint reuse depending on genome resolution

**DOI:** 10.1186/1471-2105-12-S9-S1

**Published:** 2011-10-05

**Authors:** Oliver Attie, Aaron E Darling, Sophia Yancopoulos

**Affiliations:** 1 Department of Infectious Diseases, Mount Sinai School of Medicine, NY, NY 10029, USA; 2 Department of Computer Science, University of Wisconsin-Madison, Madison, WI 53706, USA Genome Center, University of California-Davis 451 E Health Sciences Dr. Davis, CA 95616, USA; 3 Chiorazzi Lab, Feinstein Institute for Medical Research, Manhasset, NY 11030, USA

## Abstract

**Background:**

During evolution, large-scale genome rearrangements of chromosomes shuffle the order of homologous genome sequences ("synteny blocks") across species. Some years ago, a controversy erupted in genome rearrangement studies over whether rearrangements recur, causing breakpoints to be reused.

**Methods:**

We investigate this controversial issue using the synteny block's for human-mouse-rat reported by Bourque *et al*. and a series of synteny blocks we generated using Mauve at resolutions ranging from coarse to very fine-scale. We conducted analyses to test how resolution affects the traditional measure of the breakpoint reuse rate*.*

**Results:**

We found that the inversion-based breakpoint reuse rate is low at fine-scale synteny block resolution and that it rises and eventually falls as synteny block resolution decreases. By analyzing the cycle structure of the breakpoint graph of human-mouse-rat synteny blocks for human-mouse and comparing with theoretically derived distributions for random genome rearrangements, we showed that the implied genome rearrangements at each level of resolution become more “random” as synteny block resolution diminishes. At highest synteny block resolutions the Hannenhalli-Pevzner inversion distance deviates from the Double Cut and Join distance, possibly due to small-scale transpositions or simply due to inclusion of erroneous synteny blocks. At synteny block resolutions as coarse as the Bourque *et al*. blocks, we show the breakpoint graph cycle structure has already converged to the pattern expected for a random distribution of synteny blocks.

**Conclusions:**

The inferred breakpoint reuse rate depends on synteny block resolution in human-mouse genome comparisons. At fine-scale resolution, the cycle structure for the transformation appears less random compared to that for coarse resolution. Small synteny blocks may contain critical information for accurate reconstruction of genome rearrangement history and parameters.

## Background

Genomes of related organisms have been shown to share long tracts of homologous DNA sequence ("synteny blocks") across species [1]. During the course of evolution, large-scale genome rearrangements of chromosomes shuffle the order of such homologous segments. Some years ago, a controversy erupted in genome rearrangement studies over whether rearrangements are likely to recur in regions known as rearrangement hotspots [2]. Pevzner and Tesler [3] inferred the existence of such “fragile sites”, from high values of their breakpoint reuse rate (BRR). An argument by Sankoff and Trinh [4] intended to show that loss of breakpoint usage information occurs via synteny block removal, and leads to the inference of an artificially inflated breakpoint reuse rate. That argument was criticized by Peng *et al.*[5] for its flawed synteny block generation method. Subsequently, by devising a new approach for computing the BRR, Bergeron *et al.*[6] observed that rearrangement scenarios maximizing operations involving rearrangements of telomeres using only single cuts could result in much lower rates of reuse. This approach resulted in bounds for the human-mouse transformation which not only demonstrated that much lower rates are possible, but also that the BRR is extremely sensitive to genome representation and rearrangement model.

In the current paper, we reconsider aspects of the original breakpoint reuse debate. We address a theme that David Sankoff suggested in his 2006 PLoS commentary [7] was not yet fully confronted. In that commentary, Sankoff argued that high values of inferred breakpoint reuse may result from noise introduced by imprecise synteny block construction rather than due to actual genome rearrangements in the course of evolution. Imprecise synteny block construction or other processing might effectively randomize the information needed to reconstruct rearrangement history. While others use a variety of methods to explore the level of this "randomization", such as distance in Xu *et al.*[8], the number of conserved adjacencies [9], or the structure of common intervals [10], we will use the cycle structure of the adjacency graph as an indicator of "randomization". We examine how the scale of synteny block resolution affects breakpoint reuse.

To this end, and to continue the discussion of whether elevated values of breakpoint reuse can be used to infer that genome rearrangements repeatedly strike the same “hot spots” during the course of mammalian evolution, we evaluated the traditional breakpoint reuse statistic using actual data, in the context of rearrangement model and resolution for two data sets: 3-way synteny blocks for human-mouse-rat constructed by Bourque *et al.*[11], and a series of blocks generated by the Mauve genome alignment system [12] with resolutions ranging from fine-scale to coarse.

### Genome rearrangment transformations

The rearrangement transformation between two genomes with no insertions, deletions or duplications can be specified by the connections between corresponding syntenic segments in the two genomes. We call such segments *genes* even if they don't actually consist of single genes. The *adjacency graph* introduced by Bergeron *et al.*[*13*] is an elegant representation of the genomic transformation. Vertices of the adjacency graph are either the connections or *adjacencies* between two gene ends in the initial and target genomes, or solo *telomeric* gene ends at the ends of chromosomes. The complete set of adjacencies {{a_1_,a_2_}, {a_3_,a_4_}, …, {a_2N-1_,a_2N_}} specifies a genome of *N* genes. A *breakpoint* represents a disruption of their order, an adjacency of two gene ends associated in one genome but not the other.

**Figure 1:**
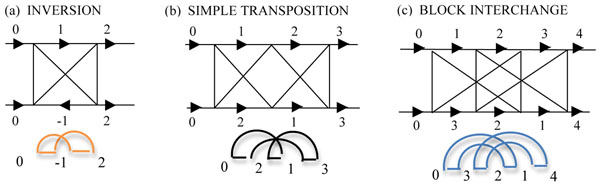
**Adjacency and breakpoint graphs for 2, 3-cycles and block interchanges** In this representation the genome graphs are superimposed over the adjacency graphs. (a) An inversion has 2 breakpoints in each genome, a DCJ distance of 1 and a breakpoint reuse rate of 2d/b=1. (b) A simple transposition with d_DCJ_=3-1=2 and b=3 has an inversion-based breakpoint reuse of 4/3. In (c) we note that a block interchange has two overlapping 2-cycles. Performing a DCJ about the first creates a circular intermediate (CI) and about the second, the CI is reabsorbed. Since b=4 and c=2 the total DCJ distance is 4-2=2. Breakpoint reuse for a block interchange in the DCJ paradigm is r=2d_DCJ_/b=4/4=1, since in the creation of the CI and its reabsorption, no breakpoints are reused in general. As for simple transpositions, the join creating the CI occurs in the same position as the subsequent cut, so one breakpoint is reused which may not be what occurs in nature! Below the adjacency graph for each example is the corresponding *breakpoint graph*. Transpositions and block intechanges look deceptively simple in their adjacency graphs. Although a block interchange appears to contain two simple 2-cycles, they are in fact made of unoriented arcs in the breakpoint graph, and similarly for the simple transposition. As a result both simple transpositions and block interchanges contain one hurdle, which increase the HP distance by 1 relative to the DCJ distance. Hence the breakpoint reuse rate for simple transpositions in the HP paradigm is r_GRIMM_=2d_HP_/b=2 and for block interchanges it is 2d_HP_/b=2*3/4=3/2.

A number of methods have been used to evaluate the minimum number of genome rearrangement events, known as *rearrangement distance*, of a genome transformation; we will focus on two: the Hannenhalli and Pevzner "HP" formulation [14,15] which involves generalized inversions, (inversions (Figure 1a), translocations, fissions and fusions), and the *double cut and join* (DCJ) [16] which includes simple transpositions (Figure 1b) and generalized transpositions (Figure 1c) in addition to the operations considered by HP.

In the adjacency graph (Figure 1), adjacencies of one genome are connected to those of another by lines representing their common gene ends. Followed continuously these lines resolve the adjacency graph into alternating closed paths called *cycles* and other continuously connected open *paths*. The ends of open paths belong to gene ends at the ends of chromosomes called *telomeres*. In our formulation of the DCJ paradigm all open paths are closed by a capping procedure described more extensively in [16-18] (for examples, see Additional File 1); essentially, adjacencies containing telomeric gene ends of chromosomes are "capped" by artificial gene ends called "endcaps". Paths that start and end on the same genome are closed by a double capped “null chromosome” on the opposite genome; paths starting and ending in different genomes are closed by connecting the two capped ends.

### Semantics, conventions, and cycle nomenclature

Due to the addition of null chromosomes, when paths are closed into cycles, both genomes artificially contain the same number of chromosomes. Some DCJ operations involve "artificial cuts" between a telomeric gene end and an endcap. In other formulations [6,13], paths are not closed hence such artificial operations do not occur. Another outcome of the device of introducing caps and closing paths is the consequent increase in breakpoints. Some formulations [3,11] count only "*internal breakpoints*" which do not include "*external breakpoints*" such as capped telomeric gene ends, or null chromosomes. In our approach we include all breakpoints, internal and external; hence telomeric adjacencies are counted.

In a departure from the usual convention in the genome rearrangement literature, we call a cycle containing *j* adjacencies in only one genome a *j-cycle* as this corresponds better to the analogous cycles for unsigned permutations. Usually it is called a *2j-cycle*. In our formulation, *1-cycles* are not counted in the DCJ distance (see below) as they are the identity transformation of a single adjacency transforming to itself; the cycle count "*c"* counts 2-cycles (usually called 4-cycles) or higher.

### DCJ operation, distance, and comparable HP distance

The double cut and join (DCJ), is a universal operation that subsumes many biological rearrangement operations.To resolve the transformation, a DCJ is performed by breaking connections in two adjacencies in the current genome and swapping gene ends so that at least one of the resulting adjacencies exists in the target, decreasing the distance by 1. Performing a single DCJ decreases the distance by 1. Each cycle can be resolved independently by DCJ. For the jth cycle containing *b_j_* breakpoints, this results in *d_j_* = *b_j_* – 1 steps*.* As all quantities are additive over individual cycles, the total DCJ distance can thus be obtained from a cycle decomposition of the adjacency graph. That is, , , and , where the sums are over cycles. Hence, the resulting DCJ distance between any two genomes can be expressed as:

*d_DCJ_* = *b* – *c*

where *b* is the number of breakpoints and *c* the number of cycles [16]. In the Hannenhalli and Pevzner formulation which is based solely on generalized inversions, the distance is:

*d_HP_* = *b* – *c* + *h* + *f*

where *b* and *c* (using our conventions) are as above, and *h* and *f* are respectively *hurdles* and *fortresses*, positive permutations which don't contain any inversions by DCJ, or require "extra inversions" to resolve by HP, and are generally rare [19]. The difference in the distance between the two formulations, *d_DCJ_* – *d_DCJ_* = *h* + *f*, consists of these relatively rare obstructions*.* Figures 1b and 1c illustrate how hurdles increase distance in the HP over the DCJ paradigm.

### Definition of the classical inversion-based breakpoint reuse rate (BRR)

In considering an “undisturbed” stretch of genome, performing a reversal creates at most two breakpoints at each step. Pevzner and Tesler [3] defined the inversion based breakpoint reuse rate (BRR) as *r* =*2d/b*, with *d* the genomic distance, and *b* the number of breakpoints*.* This "traditional" reuse statistic is *1* for reversals as long as no breakpoints are reused. The cycle distribution of such a transformation consists entirely of *2-cycles* in our nomenclature (or *4-cycles*, by the usual convention) such as in Figure 1a. If breakpoints are reused, the statistic can be as high as 2.

### BRR for (generalized) transpositions is model dependent

To see the effect on the genome for these operations, Figures 1b and 1c show adjacency graphs superimposed on their genome graphs for a simple transposition and a block interchange (BI). Both are 2-step DCJ operations which exchange two segments in a genome by creating an intermediate circular. The simple transposition reuses the same cut in the circular whereas the BI does not. A simple transposition is a special case of a block interchange. As both operations involve the creation and reabsorption of *circular intermediates* (CI) they are considered *generalized transpositions*

A *breakpoint graph*[20] (see Figure 1 for illustrative examples shown below their adjacency graphs) is a representation dual to the adjacency graph such that vertices in the adjacency graph, are lines or arcs in the breakpoint graph. Horizontal lines ("black lines") are typically adjacencies in the current genome, while arcs are "desired" adjacencies in the target. Vertices in the breakpoint graph correspond to gene ends, and are lines in the adjacency graph. Particularly for block interchanges, the creation of CI, is not easily discerned from the adjacency graphs as they look like graphs for a pair of overlapping 2-cycles which seem deceptively like inversions. Breakpoint graphs maybe more informative for identifying transpositions.

To see why this is so, we note that in the breakpoint graphs for the simple and generalized transpositions, the arcs are "*unoriented*" unlike arcs composing the breakpoint graph of the inversion in Figure 1a, which are "*oriented*". Oriented arcs have their black lines pointing in the same direction relative to the arc, whereas unoriented arcs both point away or towards the central arc. An arc's orientation is only defined for arcs rooted in the same chromosome, otherwise we consider them *non-oriented*. DCJ performed on oriented arcs produce inversions, while DCJ performed on unoriented arcs produce CI. In order for CI to be reabsorbed, the arc about which we perform the DCJ must overlap others in the breakpoint or adjacency graph.

As shown in the legend for Figures 1b and 1c, cycle structures resulting in generalized transpositions in the DCJ paradigm do not result in the same distance in the HP paradigm, in which only generalized inversions can be performed and not the creation of a CI. As a result, not only do the distances differ, but also the inferred BRR. We note that while Figure 1b shows an unoriented 3-cycle, 3-cycles also exist which contain oriented arcs. Such 3-cycles can be resolved by inversions. For oriented 3-cycles, DCJ and HP distances agree as does breakpoint reuse.

### Random permutations, cycle structure, and BRR for longer cycles

If we continue performing reversals indefinitely in the same stretch of genome, eventually we will start to reuse breakpoints (unless the simplifying assumptions of the “Infinite Sites Model” of Ma *et al. *[*21*] are used, which prevent breakpoint reuse). Consequently, the cycle structure will change. Each reuse of a breakpoint increases the length of the cycle in which that breakpoint appears. Longer and longer cycles appear which continue growing in number and length. The resulting permutation will exhibit increased breakpoint reuse. We see this by computing the inversion based breakpoint reuse for a longer cycle having *n* breakpoints. The DCJ distance for a cycle containing *n* breakpoints is *n-1* and, as we have seen, is identical to the HP distance unless there are hurdles and fortresses. The inversion based BRR statistic is therefore *r_longcycle_* = 2*d*/*b* = 2(*n* – 1)/*n*, which approaches 2 as *n* gets large. Since distance is additive over cycles, and so are breakpoints, for transformations containing *N* such cycles the total BRR becomes, *r_longcycle_* = 2*N*(*n* – 1)*/Nn* = 2(*n* – 1)/*n* → 2 for large *n*.

As more and more random operations are performed between two genomes, they become increasingly divergent and randomized relative to one another. This affects the cycle structure. Considering results from a paper on random signed gene order permutations by Sankoff and Haque [22]which constructed cycles of the breakpoint graph of random permutations, Richard Friedberg communicated results on cycle structure to us which we discuss in the next section. In particular, he shared a result for the expected number of *j*-cycles in the random edge graph for transformations by DCJ for genomes containing only circular chromosomes. As gene number per chromosome gets large, we surmise results for the *unrestricted circular case* (allowing intermediate genomes to contain circular chromosomes) coincide with those for the *restricted linear case* (circulars are immediately reabsorbed).

### Cycle structure of random unsigned permutations

Before discussing the signed permutation case in the next section, we first consider the cycle structure of unsigned permutations (permutations of objects without consideration of orientation). The results for the unsigned permutation case have already been reported [23] . These formulas are analogous to those for signed permutations. In the unsigned case, the expected number of *j*-cycles is:(1)

To compare this later with the result for signed permutations, we write it as:

We can get the expected number of cycles directly by summing eq (1) over *j*:(2)

Unfortunately the analogous formula for bipairings (ie signed permutations of genes with orientation) does not have the simplicity of eq (1), so we cannot accomplish this as easily for signed permutations. Nor can we compute *c* by summing an approximate formula because its error becomes large when *j* is near *N*, no matter how large *N* is. Fortunately, there is an indirect way of deriving eq (2) bypassing eq (1) so that *c* is found without finding the individual values of *q_j_* and the result agrees with eq (2). This indirect way has the merit that it also yields the variance of *c*, which cannot be inferred from eq (1). With a slight modification this method also works for bipairings. It yields:

<*c*> = 1 + 1/3 + 1/5 + … (2*)

Now these individual terms do not correspond to the formula for signed permutations, eq (1*) below. For small *j* the *j*th term of (2*) is larger than *q_j_*, but for *j* near *N* it is smaller. To solve for  one can write:

<c_3_> = <c> – (<*q*_1_> + <*q*_2_> + <*q*_3_>) (3, 3*)

true for both (signed and unsigned) problems. In the unsigned permutation problem this simplifies to

<c_3_> = 1/4 + 1/5 + 1/6 + …

which has no analogue for bipairings. To evaluate (3) for bipairings one must use (2*) for <*c*> and approximate formulas for the equivalent terms <*q*_1_>, <*q*_2_>, <*q*_3_>.

### The cycle structure of random signed genome permutations

In the case of signed genome permutations, 2*N* gene ends can be paired into *N* adjacencies in (2*N* – 1)!! different ways where K!! is a *double factorial* and is equal to K(K-2)(K-4)…k (with k=1 if K is odd, and 2 if K is even). The comparison of two such pairings permits a cycle structure to be constructed. For an adjacency graph with *N* adjacencies in each genome, let *q_j_* be the number of j-cycles and  the total number of cycles.

• Then for such a random adjacency graph:

For large N and small j the asymptotic limit of equation (1*) is:

which can be compared to the analogous result for unsigned permutations, eq (1).

Thus for signed permutations:

• The expected number of cycles, by the indirect method mentioned previously is:

where γ=Euler’s γ= 0.577. Hence, for large *N* we have that <*c*> is given approximately by:

• The expected number of cycles of length > j is:

<*cj*>=<*c*>*–* <*q*_1_> – <*q*_1_> –…–<*q_j_*>

which can be evaluated from (1*) and (2*) and approximately from (1a*) and (2a*).

We wish to know <*c*_3_>. Thus, for large *N*,

<*c*_3_> = <*c*> – (<q_1_> + <q_2_> + <q_3_>) (3*)

## Methods

### Summary of our approach

To explore whether randomization of genome transformations occurs beyond that due to actual evolutionary events recorded in the "signal in the genome" we have adopted the traditional breakpoint reuse statistic. We feel this statistic serves as an indicator of overall apparent breakpoint reuse and best reflects the approach followed by the pioneering paper of Pevzner and Tesler (2003). Towards this end, we consider the traditional inversion-based breakpoint reuse rate in three data-based computational experiments discussed in the next three sections. We calculated BRR for the following [h=human, m=mouse, r=rat]:

1) a series of Mauve synteny block sets at resolutions ranging from fine to coarse

2) the 300 kb Bourque *et al* (2004) h-m 3-way h-m-r blocks with block removal

3) the 141min LCB Mauve blocks (defined below) before and after block removal

### Generating synteny blocks with mauveAligner

We applied the "mauveAligner" from the Mauve genome alignment system [12] to search for high-resolution 3-way locally collinear blocks (Synteny Blocks) among the genomes of Mouse, Rat, and Human. Mauve uses a seed-and-extend local multiple alignment method to identify high-scoring local alignments, which it then clusters into locally collinear blocks—groups of matches in the same order and orientation among each genome. We assign each locally collinear block (LCB) a weight *w*(*B*) equal to the sum of lengths of the constituent ungapped local alignments. The weight of a block *w*(*B*) can be formally defined as , where a block *B* consists of one or more ungapped local alignments *m*, and *length*(*m*) is the number of nucleotides covered by *m*. In the context of mauveAligner, the matches in a block must be in the same order and orientation in all genomes. That is, blocks are internally free from rearrangement.

Because mauveAligner generates local alignments in unique regions of sequence only, it typically generates few LCBs containing only repetitive regions, and any LCBs generated in such regions tend to be small, having a low weight. mauveAligner filters out those spurious LCBs by iteratively removing low-weight LCBs in a process called greedy breakpoint elimination [12]. Greedy breakpoint elimination is analogous to iterative block removal using *w*(*B*) to give an ordering for blocks to be removed. At each step in the process, the lowest weight LCB is removed. If one or more rearrangement breakpoints have been eliminated, any surrounding LCBs may coalesce with each other. The weight of a coalesced LCB is equal to the sum of weights of LCBs that were joined.

In the present work, the RepeatMasked assemblies of human (NCBI 35), mouse (NCBI 33), and rat (RGSC 3.4) were searched for unique 3-way seed matches on the forward and reverse strands using the palindromic seed pattern: 11111*111*11*1*11*111*11111 [24]. Initial seed matches were maximally extended in each direction until the seed pattern no longer matches at any overlapping position. A total of 922,081 ungapped 3-way local alignments containing unique sequence resulted. These initial matches have a minimum length of 27 (dictated by the seed pattern above). The initial set of 3-way matches gave rise to 567,782 LCBs, to which we applied greedy breakpoint elimination to remove all LCBs up to a minimum weight of 55, yielding a baseline set of 520,423 3-way matches that compose 6483 LCBs. The minimum sequence alignment length among these blocks is 55 nucleotides. We further applied greedy breakpoint elimination to the baseline set of 6,483 LCBs, recording the observed genomic permutation at each successively higher LCB weight up to a minimum weight of 100,000. At minimum weight 97,673 (the last weight before 100,000), there are 75 3-way LCBs among the mouse, rat, and human genomes. At weights larger than 500, the LCB weight is approximately proportional to the overall chromosomal span of an LCB, with chromosomal span 100-1000x the LCB weight. The data set is included as Additional Files 2,3,4,5,6,7,8,9,10

### Calculating genomic distance by GRIMM and DCJ

The HP genomic distance for the Mauve synteny block sets constructed at each successive minimum LCB weight was computed using the GRIMM [25] server. We computed the DCJ [16] distance using a C++ program which inputs the genome as a signed permutation (exported from Mauve) as the GRIMM server does, and outputs the DCJ distance, the number of breakpoints, the number of cycles of each kind and their total, and the number of null chromososmes.

## Results

### Onset of transpositions for human-mouse (h-m) rearrangements

**Figure 2:**
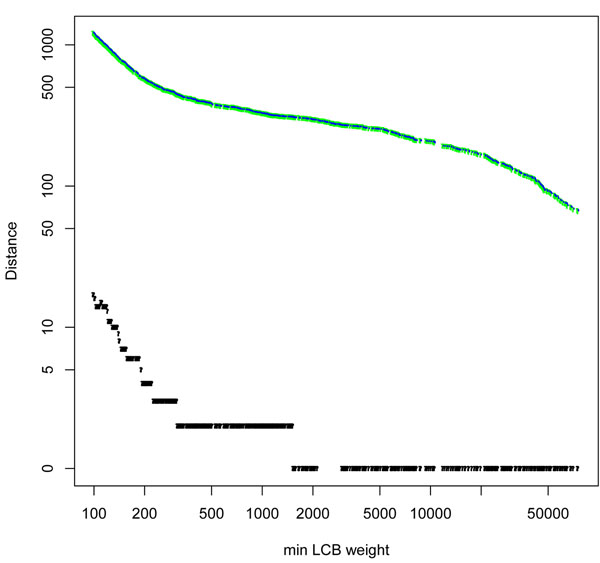
**Distance and hurdles over the full range of Mauve synteny block resolution** The HP (blue) and DCJ (green) distances are virtually indistinguishable, their difference for h-m is shown in black. The distances far exceed their difference.

We found that the HP and DCJ distances agree identically at low resolution but at high resolution, the GRIMM distance exceeds the DCJ distance. In Figure 2, we display these distances on a log scale so as to simultaneously display the difference between GRIMM (HP) and DCJ values. The maximum GRIMM (HP) value is 1223 while the maximum value of the DCJ distance is 1206. The maximum difference between these two distances is therefore relatively small, only 17 or 1.4% of the total (HP) distance at highest resolutions.

We assume the differences we found between GRIMM (HP) and DCJ distances are due to hurdles, since *d_GRIMM_ - d_DCJ_* = *d_HP_ - d_DCJ_* = *h* +*f* where fortresses, in breakpoint graphs containing an odd combination of hurdles, are extremely rare. The hurdles in our human-mouse (h-m) transformations may be due to simple transpositions and generalized transpositions (GT) as in Figures 1b and 1c, which differ in HP and DCJ distances as shown. GT onset in Figure 2 occurs at min LCB weights 2153 for h-m corresponding to a DCJ distance of 293 and GRIMM of 294

### BRR for human-mouse Mauve blocks at different resolutions

The traditional inversion based breakpoint re-use rate, *2d/b*, where *d* is the genomic distance and *b* the number of breakpoints [5] was computed for both GRIMM (HP) and DCJ distance measures on each set of Mauve synteny blocks. The two distances are identical for most of the min LCB weight range represented by Mauve block sets (Figure 2), and the calculated breakpoint reuse rate would also be the same in that range if the number of breakpoints agreed for both GRIMM and DCJ distances. However, to follow the 2003 paper of Pevzner and Tesler in PNAS [3], we used "internal" breakpoints to calculate the GRIMM breakpoint reuse rate. For the DCJ BRR calculation we used "internal + external" breakpoints.

**Figure 3:**
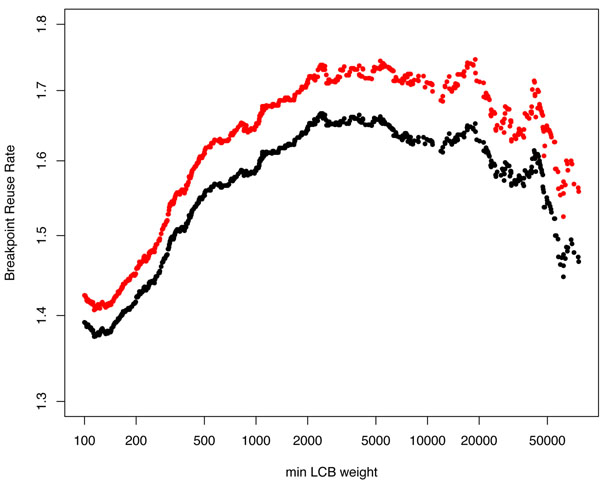
**BRR as a function of min LCB weight** Differences between the GRIMM(HP) and DCJ BRR values are due to the use of *internal* breakpoints for the GRIMM measure and *internal*+*external* breakpoints for the DCJ measure. Typical differences are of the order of 0.05 between these measures, but both measures deviate as much as 0.35 from their respective maxima.

**Figure 4:**
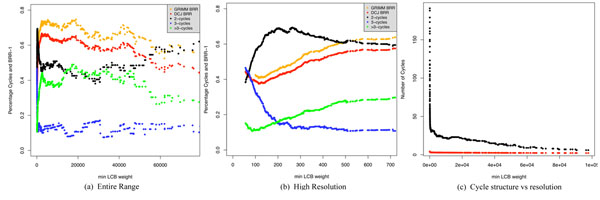
**Cycle structure and BRR as a function of LCB weight** In this figure, cycle structure is compared with breakpoint reuse rate (BRR). The entire range of min LCB weight is shown in (a). In (b) we magnify the high resolution (low min LCB weight) portion of the graph. In (c) we represent the number of >3-cycles (usually called >6-cycles) vs the theoretical prediction for random permutations derived earlier. Cycle decomposition of the human-mouse transformation is graphed against BRR minus 1 so that the contributions of different cycles can be evaluated. The Grimm breakpoint reuse rate minus 1 is shown in mustard orange in (a) and (b). The corresponding DCJ version is in red in the same two panels. The fraction of 2-cycles (conventionally 4-cycles) are shown in black in (a) and (b). The fraction of 3-cycles (usually called 6-cycles) and >3 cycles is shown respectively in blue and green in (a) and (b) and in black in (c).

Even though the DCJ and HP distances are largely identical over most of the range, the graphed values of BRR we present in (Figures 3, 4a, 4b) using the HP and DCJ distances we evaluated are not the same. They run essentially parallel to each other. Their difference is not due to the difference in distance for the most part (except at highest resolution where the effect due to the difference in distance is nondetectable), but rather is due to the manner of evaluating breakpoints.

Our maximum values for the BRR range from 1.67 (for the "DCJ" value) to 1.738 (for the "HP" value of BRR) depending on whether the number of breakpoints was evaluated using external breakpoints in addition to internal breakpoints (for the DCJ value) or only internal breakpoints (for the HP value). This variation is consistent with the range of values for BRR in [3] (which goes from 1.633 to 1.9).

The lowest BRR we observed is 1.376 (for the DCJ version), occurring in Mauve synteny blocks with min LCB weight 141. These blocks have an average chromosomal length of 2.66 Mb, a GRIMM distance of 813, and the total number of blocks is 1143. Increasing the min LCB weight from here (and hence decreasing the synteny block resolution), we observe a dramatic rise in both breakpoint reuse curves, which reach their maximum at min LCB weight 2386 (Figure 3). The HP BRR then plateaus and stays in the same range until min LCB weight 5518 (Figure 3). It remains over 1.7 for min LCB weights up to 40000, which have average block size 22.7 Mb, for a total of 133 blocks, and a GRIMM distance of 116.

### Dependence of BRR and cycle structure on resolution

To understand the variation in the breakpoint reuse measures based on synteny block resolution, we calculated the cycle structure for the human mouse transformation on a series of progressively increasing resolution Mauve synteny blocks. Figure 4 shows the behavior of the breakpoint reuse measures along with the proportion of cycles for 2, 3 and >3 cycles for human-mouse as they vary with LCB weight.

To interpret how these fractional cycle curves affect the BRR, we wish to express the breakpoint reuse rate BRR *r* for the entire transformation as a weighted sum over the BRR of individual cycles *r_j_*, that is,(3)

where we wish to determine the coefficient *c_j_* in such a way as to involve the fractional distributions over kinds of cycles. Since the total breakpoint reuse is r = 2*d*/*b*, where both the total distance and the total number of breakpoints are summed over cycles, that is  and  hence the coefficient for equation (4) is:

*c_j_* = *b_j_*/*b*.

However, the sum in equation (4) is over individual cycles, and not kinds of cycles. To accomplish this we group cycles of the same kind together. If *n_k_* is the number of k-cycles the new sum over kinds of cycles, ie 2-cycles, 3-cycles, 4-cycles, etc is:

where *b_k_* is the number of breakpoints in a k-cycle. Expressing the coefficient in fractions of cycles, (2-cycles, 3-cycles etc) with the fraction of k-cycles =*f_k_*=*n_k_/Σn_k_*_._ and the sum over *n_k_*, that is, *Σn_k_*=*c*, the total number of cycles, we arrive at a weighted sum over different kinds of cycles:

where *b_k_* is the number of breakpoints in a k-cycle, *f_k_* the fraction of k-cycles and *r_k_* is the *BRR* of a k-cycle, or *r_k_* = 2*d_k_*/*b_k_* = 2(*k* – 2)/*k*.

### Resolution based cycle structure for human-mouse

In Figure 4 we represent the dependence of the cycle structure on min LCB weight. Figure 4a shows the dependence of the cycle structure over the full range of min LCB weight. Figure 4b, shows the high-resolution end. At high resolution, the dependence of BRR bottoms at min LCB weight 141 approximately where the proportion of 2-cycles (usually called 4-cycles) is at a maximum and the proportion of >3-cycles (usually called 6-cycles) are at minimum. At the minimum of breakpoint reuse, 64% of the cycles are 2-cycles, 23% are 3-cycles and 12.5% are cycles of length greater than 3. At highest resolutions (below min LCB weight 140) a rise in the 3-cycle and >3-cycles fraction and corresponding drop in the 2-cycles produces a rise in BRR.

In the plateau, nearly half (45% to 48%) of the cycles are 2-cycles, 11.26 to 12.5% of the cycles are 3 cycles, and 39 to 43.66% of the cycles are of length greater than 3. The initial rise to the plateau from the minimum of breakpoint reuse corresponds to a rise in the percentage of 2-cycles, a decline in the percentage of 3-cycles and in the percentage of cycles of length greater than 3. The decline in breakpoint reuse as the min LCB weight grows is due to a rise in the percentage of 2-cycles from 48.5% at minimum LCB weight 51235 to 67% at LCB weight 97673 while the percentage of greater than 3-cycles declines from 36.4% to 22.2%.

The decline in breakpoint reuse as min LCB weight grows past 50000 is due to finite chromosome length. Entire chromosomes can be spanned by Mauve synteny blocks at their lowest resolution. The average length of blocks with highest min LCB weight (100,000) is 42.4 Mb, almost the size of the smallest (human) chromosome, 46.9 Mb. At such resolutions, the BRR diminishes, the percentage of 2-cycles rises and that of >3 cycles declines.

### Minimum of BRR and cycle structure

The region around the minimum of BRR is very interesting: near min LCB weight 200, the fraction of 2-cycles peaks (Figure 4a and b) and the fraction of >3 cycles is at a minimum at a slightly lower min LCB weight than the min of BRR. It is in this overall region that the transformations are least complicated, and the "signal in the genome" is best preserved. There are mainly "simple generalized inversions" since we are simultaneously at the maximum of the 2-cycles fraction and at the min of the >3 cycles, hence at the smallest fraction of long cycles, although there is a caveat in that longer cycles have correspondingly higher weights. We next compare our computed cycle structures for human-mouse at different resolutions with the previously derived predictions of cycle structure for random permutations.

### Low resolution cycle structure approaches random permutation

We compared the total number of >3 cycles in the human-mouse transformation to the expected distribution for a random permutation having the same number of synteny blocks derived earlier. As resolution decreases, e.g. with increasing min LCB weight (Figure 4c), the number of >3 cycles of the transformation approaches the theoretical distribution derived earlier for this number in a random permutation having the same number of synteny blocks.

### Systematic block removal for the 300 kb h-m Bourque *et al.* blocks

Bourque *et al. *[11], constructed 3-way synteny blocks for human, mouse and rat, using GRIMM-Syntenywhich can be accessed online [3,5]. GRIMM-Synteny preserves information about microrearrangements within synteny blocks, dividing the blocks into “micro” and “macro” by a choice of parameters. Choosing 300 kb as their cutoff, they arrived at 394 blocks for human-mouse.

We applied the following protocol of successive block removal on these 300 kb “Bourque” blocks. Blocks were removed in a stepwise fashion, starting with the smallest blocks. Remaining blocks were concatenated if they appeared in the same orientation and order in both genomes. Although our approach follows the spirit of the Sankoff-Trinh [22] block removal procedure, in their approach, blocks were removed from simulated genomes. Ours used a set of blocks that were derived from real data. Theirs were generated so as to have no breakpoint reuse. After removing blocks, the Sankoff-Trinh procedure amalgamated the proximal blocks in a manner subsequently shown to be faulty [5]. Since their data was simulated as a permutation file, it had no actual size. Since we used real data, the blocks we used have size and orientation.

We calculated breakpoint reuse after each set of blocks was removed, and plotted the resulting curves versus the size of blocks removed (Figure 6). BRR was calculated using GRIMM [25] via the Hannenhalli and Pevzner algorithm [14,15] and via DCJ [16] with breakpoints evaluated as previously discussed. As before, the differences in these values of breakpoint reuse are not significant, but are mainly due to the different methods of counting breakpoints (*internal* for GRIMM and *internal*+*external* for DCJ). The change in breakpoint reuse with block removal (for either BRR measure) for the Bourque *et al*. blocks is not significant. As the breakpoint reuse rates do not change significantly with block removal, we hypothesize that this situation is similar to the plateau region in Figures 3 and 4a and 4b where the genomic permutation is "randomized". In the next sections we perform the same block removal procedure on the Mauve blocks most closely corresponding to the 300 kb Bourque *et al.* blocks.

**Figure 5:**
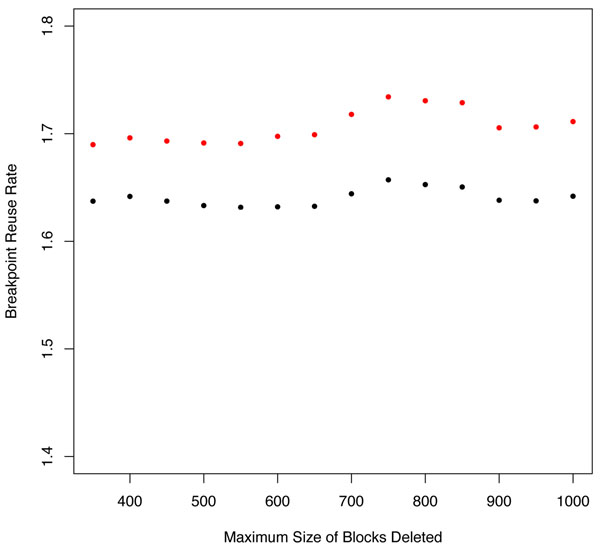
**Dependence of BRR on maximum size of deleted blocks for Bourque *et al* synteny blocks** GRIMM or HP (red) and DCJ (black) breakpoint reuse values for Bourque *et al* synteny blocks are in league with the maximum values previously obtained for resolution dependent Mauve synteny blocks. The elimination procedure does not affect the BRR values significantly.

**Figure 6:**
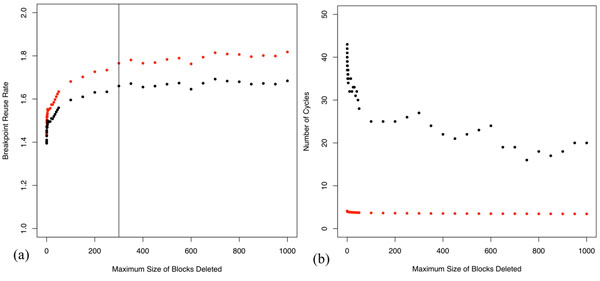
**Block elimination and BRR and the number of >3 cycles for the Bourque *et al*-like Mauve blocks vs those from a random permutation** (a) GRIMM or HP (red) and DCJ (black) BRR values initially rise with block removal however after blocks smaller than 300 kb are removed the curves plateau. (b) The number of >3 cycles (black dots) approach those (in red) of the derived random permutation matched for number of synteny blocks as blocks are removed starting with the smallest.

### Corespondence between Mauve blocks and "Bourque-*et al.*" blocks

No direct correspondence can be made between the Mauve synteny blocks and those of Bourque et al 2004, since Mauve blocks and the Bourque *et al*. 3-way synteny blocks for human mouse rat were generated from different genome assemblies. By comparing various parameters including GRIMM and DCJ distance, breakpoints, and Breakpoint Reuse Rate (BRR), we derived a rough correspondence (Tables 1 and 2); the Mauve blocks with min LCB wt 141 most closely correspond to both the 300 kb and 1 Mb Bourque *et al*. blocks. We call these the *Bourque et al.-like Mauve blocks*.

**Table 1:** **Comparison of Bourque 300 kb blocks with Mauve** The table shows the correspondence between the Bourque 300 kb blocks and the Mauve blocks. The closest corresponding minimum LCB weight is in parentheses.

	Bourque 300 kb	Mauve (closest LCB)	Mauve w/out removing blocks	Mauve after removing <300 kb blocks
**#Synteny blocks**	394	395 (991)	378 (1096)	347 (141)
**average size (Mb)**	6.851	6.855 (1042)	7.983 (1096)	7.887 (141)
				
distance	338	338 (877)	322 (1096)	308 (141)
**GRIMM** breakpoints	417	417 (980)	384 (1096)	359 (141)
breakpoint reuse	1.645	1.646 (549)	1.694 (1096)	1.72 (141)
				
distance	338	338 (877)	320 (1096)	308 (141)
**DCJ** breakpoints	432	431 (861)	397 (1096)	371 (141)
breakpoint reuse	1.550	1.550 (471)	1.612 (1096)	1.66 (141)

**Table 2:** **Comparison of Bourque 1 Mb blocks with Mauve** This table shows the correspondence between the Bourque 1 Mb blocks and the Mauve blocks. Again the closest minimum LCB weight is in parentheses.

	Bourque 1 Mb	Mauve (closest LCB)	Mauve 1 Mb after removing < 1 Mb blocks
**#Synteny blocks**	273	273 (5889)	248 (141)
**average size (Mb)**	9.820	9.797 (3006)	10.500 (141)
			
distance	246	246 (5548)	245 (141)
**GRIMM** breakpoints	296	296 (5565)	273 (141)
breakpoints reuse	1.662	1.662 (742)	1.736 (141)
			
distance	246	246 (5548)	245 (141)
**DCJ** breakpoints	312	312 (4154)	285 (141)
breakpoint reuse	1.577	1.578 (770)	1.719 (141)

### BRR for the Bourque *et al.*-like Mauve blocks

We repeated our stepwise block removal protocol for the Bourque *et al.*-like Mauve blocks (Mauve blocks at min LCB weight of 141), since after block removal these Mauve blocks most closely resemble the 300 kb Bourque *et al.* blocks. In addition, blocks at min LCB weight 141 have the minimum observed value of BRR (see Figure 4b). Although both GRIMM and DCJ BRR curves initially rise with block removal for the min LCB weight 141 Mauve blocks, after all blocks smaller than 300 kb are removed, the curves enter a plateau (Figure 6a).

Our prior experience with the sets of Mauve blocks at different resolutions suggests that when enough smaller size blocks are removed, the remaining blocks approach a more randomized cycle structure. The BRR curves in Figure 6a for the Mauve blocks after >300 kb blocks have been removed resemble those for the Bourque *et al*. blocks (Figure 5) although the BRR values do not match exactly. The existence of the plateau suggests the Bourque *et al*. blocks have lost rearrangement information encoded by true rearrangement breakpoints and are approaching a random permutation.

### >3 cycles for Bourque et al.-like Mauve blocks with block removal

In Figure 6b we note that the divergence between the >3 cycle numbers for actual vs expected number of cycles for a random permutation is greatest when no blocks are removed and decreases with the proportion deleted. Although the two curves in Figure 6b never merge, the number of cycles greater than 3 approaches the curve depicting the expected number of >3 cycles lending credence to the notion that the permutation becomes increasingly randomized as blocks are removed.

## Discussion

### Alternative measure for breakpoint reuse

The work presented here uses the classical definition of breakpoint reuse rate. In a different approach, Bergeron *et al.* (2008) devised a new way of calculating the breakpoint reuse rate, BRR, and showed that by this definition the BRR is intimately connected to particular rearrangement scenario and model. They redefined the breakpoint reuse rate as:

*r* = *C/b*

where *C* is the total number of cuts made by the operations of the scenario, and *b* the number of B-vertices (ie in the target genome) in long cycles or paths. In methods that force an artificial closure of paths ending in telomeric adjacencies, and an equalizing of chromosome number resorting to use of null chromosomes (including our formulation of the DCJ), there is no biological basis for cuts performed between caps and gene ends or between caps and caps in null chromosomes. The traditional breakpoint reuse measure can double-count the number of actual cuts performed for each DCJ in specific scenarios, leading to a severe overestimate of the BRR. Bergeron *et al*. (2008) followed up on this insight by devising a number of ingenious manoeuvres to find scenarios that either maximize or minimize their statistic. For some long paths it is possible to decrease the number of cuts by a factor of two, thereby radically diminishing the effective breakpoint reuse. By the new definition, the value of the breakpoint reuse rate can become less than 1, achieving the following bounds for human-mouse:

0.89 ≤ r ≤ 1.51

Although the variation we achieved is not quite as dramatic, as that obtained by Bergeron *et al*. we did attain nearly half this variability just by changing the resolution of the synteny blocks.

In another work on this subject [26], Sinha and Meller investigated the relationship between BRR and synteny blocks using a simple approach to synteny block aggregation depending on two principle parameters: the maximum gap (*max_gap*) between adjacent blocks to be merged, and the minimum length (*min_len*) of synteny blocks. They found that the classical breakpoint reuse rate was almost constant for different data sets and a wide range of parameters, which roughly corresponds to our results for BRR in the plateau region (our Figures 3,4,5,6). Their work did not investigate synteny blocks of the high resolution presented here. Although they do not report the actual chromosomal span of their smallest blocks, the block set they analyzed with the highest synteny block count is generated by GRIMM and, having 2000 blocks, has much fewer than our highest resolution dataset which has about 6500. Like us, they also found that BRR was strongly correlated with HP distance, increasing with more divergent genomes. To compare our results for BRR with theirs, we calculated the minimum, maximum, mean and standard deviation of the BRR over the range in Figure 3 as well as for regions corresponding to the rise in the graph (from min LCB weight 100 to 2717), the plateau (min LCB weight 2717 to 42336), and the fall (min LCB weight 42336 to 76134). The results are summarized in Table 3.

**Table 3:** **Statistics of DCJ BRR for** Figure 3. Our graph in Figure 3 is divided into separate regions corresponding to Rise, Plateau, and Fall. The Min, Max, Mean and St Deviation of the BRR based on the DCJ are tabulated for each region separately, and for the overall graph. The Sinha-Meller results are approximate values reported in [26]

	Min BRR	Max BRR	Mean BRR	St deviation
Entire Graph	1.374833	1.6667	1.559591	0.08839528
				
Rise	1.374833	1.6667	1.525399	0.09207892
				
Plateau	1.5675789	1.664557	1.627744	0.02758151
				
Fall	1.447619	1.609023	1.531881	0.05047854
				
Sinha-Meller	1.53	1.67	~1.63	~0.025

Our results largely agree with those of Sinha and Meller for the plateau region, however, Sinha and Meller did not arrive at values of breakpoint reuse varying as much as ours did overall. Our breakpoint reuse rate varied nearly 20 % of the maximum value.

## Conclusions

High values of breakpoint reuse have been used to justify the existence of fragile sites in genome rearrangement scenarios. Although fragile sites may well exist in the course of mammalian genome evolution, we argue that computed high values of the "traditional" breakpoint reuse statistic do not yield conclusive evidence for the existence of such sites. Rather, as we have shown, the cycle structure for such high BRR transformations these genome transformations are more like random permutations. Small synteny blocks may contain critical information about rearrangement history. As small synteny blocks are lost, either by diminished resolution or block removal, the numbers of >3 cycles increasingly approach the expected values for a random permutation. For the Bourque *et al.* 300 kb blocks, block removal did not diminish BRR. In an experiment in which we best matched the Bourque blocks with Mauve (at min LCB weight 141 blocks) we showed that the behavior of BRR curves of the Mauve blocks upon block removal also had flat levels once all blocks spanning less than 300 kb were removed. We posit that blocks spanning less than 300 kb, which are missing from the Bourque *et al*. dataset, may encode vital information about the true rearrangement history and associated parameters. The distribution of the number of cycles >3 corroborates our suggestion that information is lost either with diminished resolution or with systematic block removal starting with the smallest blocks. Finally, because the GRIMM and DCJ distance was not significantly different for a great portion of the resolution range, the difference in rearrangement models did not come into play except at the highest resolutions. At these resolutions, evidence for transpositions exists but they comprise less than 2% of all rearrangements.

An implication of our study is that precise definition of synteny blocks both large and small is crucial for accurate inference of rearrangement history parameters. Small blocks matter. Although larger blocks can be predicted more reliably, homology can be confidently predicted even for small regions spanning less than 1000 nucleotides using BLAST statistics. Probabilistic methods for Synteny Block reconstruction [27] can be used to assign a confidence value (or posterior probability) to blocks large and small. Future work might investigate the relationship between filtering blocks using such confidence estimates and rearrangement parameters such as breakpoint reuse, cycle count distributions, and others.

Finally, even though we suggest that the breakpoint reuse rate may be lower than previous estimates, we note that our findings do not preclude the existence of chromosomal regions with an unusually large number of closely spaced rearrangement breakpoints or "fragile regions". Even if breakpoint reuse is low, breakpoints might cluster near each other on the chromosome. Indeed this could be a natural consequence of Nadeau and Taylor’s work: if breakpoints are selected uniformly at random along a genome, the inter-breakpoint distances will be geometrically or exponentially distributed (as will synteny block lengths), and clusters of nearby breakpoints may exist purely by chance. However, Pevzner and Tesler [3] inferred an excess of short distances exist between breakpoints over the expected exponential distribution in the random model, concluding this implied the existence of fragile regions solely based on elevated values of their breakpoint reuse statistic. Even though we contend this argument may not hold as we have shown the breakpoint reuse statistic depends significantly on the resolution scale used in the analysis, other investigators have shown the existence of breakpoint clusters [28-30].

## List of abbreviations

BI: block interchange; BRR: breakpoint reuse rate; CI: circular intermediate; DCJ: double cut and join; GT: generalized transpositions; h-m: human-mouse; h-m-r: human-mouse-rat; HP: Hannenhalli-Pevzner; kb: kilobase; LCB: locally collinear block; Mb: megabase.

## Competing interests

AED served as co-editor for the supplement, but was not involved with the review of this paper. OA and SY declare that they have no competing interests.

## Authors' contributions

SY conceived the idea of using Mauve and the DCJ algorithm to investigate the behavior of BRR based on resolution using real data. SY and AED designed the approach using Mauve for this purpose. AED generated the 3-way locally collinear synteny blocks for human-mouse-rat genomes using Mauve. SY and OA designed and implemented the DCJ program. OA wrote perl and shell scripts, and R programs to process and analyze the data. SY, OA and AED analyzed the results. SY, OA and AED wrote and edited the manuscript. All authors read and approved the final manuscript.

## Supplementary Material

Additional file 1 **Appendix on Capping Examples for the DCJ** This appendix provides illustrative examples on how capping is performed in our version of the DCJ paradigm. Click here for file

Additional file 2 **Permutation file** These files contain the permutations for the human-mouse-rat collinear synteny blocks which are output by Mauve. Click here for file

Additional files 3 **LCB files** These files contain the lcbs for the human-mouse-rat collinear synteny blocks files which are output by Mauve. In addition, there are three files giving the lengths of the chromosomes of each genome. Click here for file

Additional files 4 **LCB files** These files contain the lcbs for the human-mouse-rat collinear synteny blocks files which are output by Mauve. In addition, there are three files giving the lengths of the chromosomes of each genome. Click here for file

Additional files 5 **LCB files** These files contain the lcbs for the human-mouse-rat collinear synteny blocks files which are output by Mauve. In addition, there are three files giving the lengths of the chromosomes of each genome. Click here for file

Additional files 6 **LCB files** These files contain the lcbs for the human-mouse-rat collinear synteny blocks files which are output by Mauve. In addition, there are three files giving the lengths of the chromosomes of each genome. Click here for file

Additional files 7 **LCB files** These files contain the lcbs for the human-mouse-rat collinear synteny blocks files which are output by Mauve. In addition, there are three files giving the lengths of the chromosomes of each genome. Click here for file

Additional files 8 **LCB files** These files contain the lcbs for the human-mouse-rat collinear synteny blocks files which are output by Mauve. In addition, there are three files giving the lengths of the chromosomes of each genome. Click here for file

Additional files 9 **LCB files** These files contain the lcbs for the human-mouse-rat collinear synteny blocks files which are output by Mauve. In addition, there are three files giving the lengths of the chromosomes of each genome. Click here for file

Additional files 10 **LCB files** These files contain the lcbs for the human-mouse-rat collinear synteny blocks files which are output by Mauve. In addition, there are three files giving the lengths of the chromosomes of each genome. Click here for file
