# Supplementary material for: The rise and fall of breakpoint reuse depending on genome resolution
Source: BMC Bioinformatics. 2011 Oct 5;12(Suppl 9):S1. doi: 10.1186/1471-2105-12-S9-S1 (PMC3283316; doi:10.1186/1471-2105-12-S9-S1)
Supplement: Additional file 1 — Appendix on Capping Examples for the DCJ This appendix provides illustrative examples on how capping is performed in our version of the DCJ paradigm. [file 1471-2105-12-S9-S1-S1.pdf]

Appendix: Capping Examples for the DCJ Paradigm

These examples are taken from Yancopoulos S, Attie O, Friedberg R: Efficient sorting of genomic permutations by translocation, inversion and block interchange. *Bioinformatics* 2005, 21(16):3340-3346.

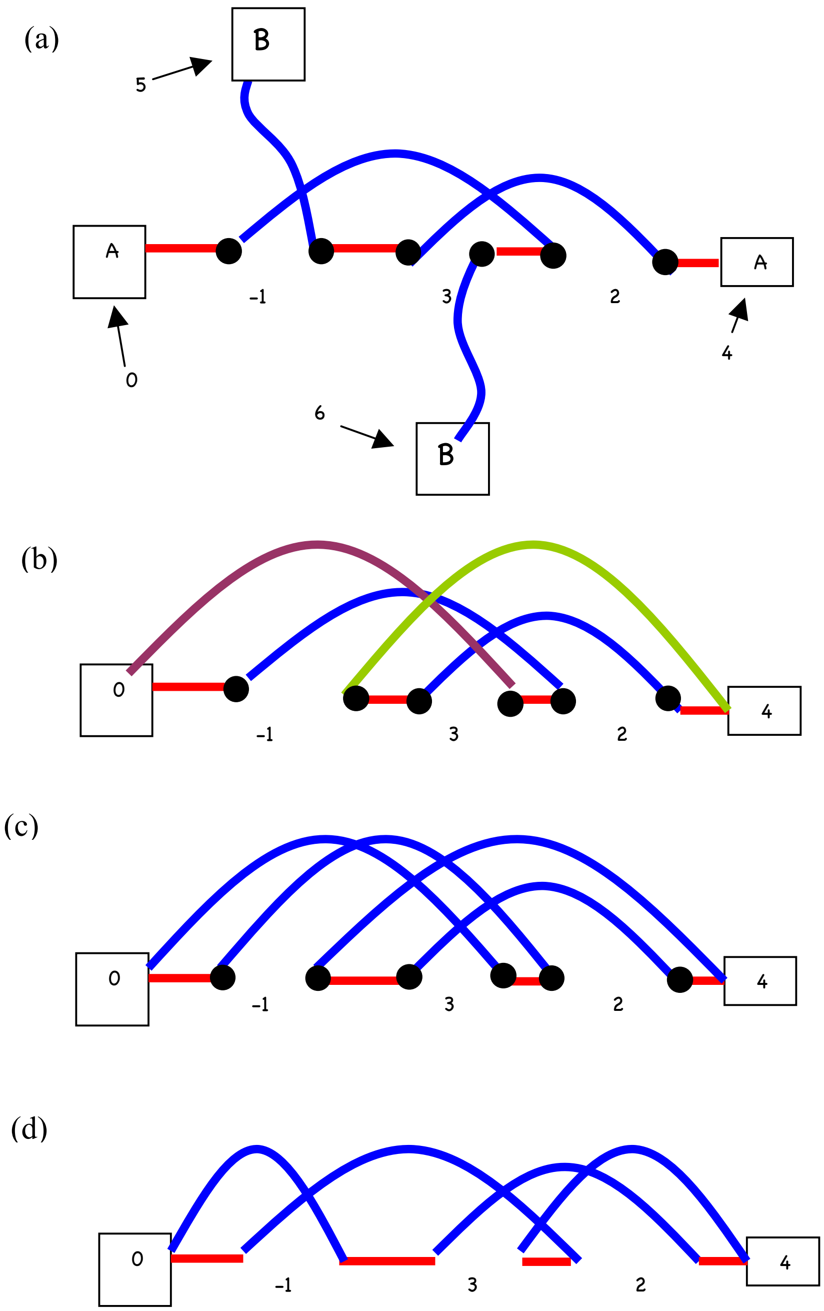

Example from Figure 7

Genome A	Genome B
-1 3 2	1, 2, 3

After capping genomes become:

Genome A	Genome B
0, -1, 3, 2, 4	5, 1, 2, 3, 6

Cap identification

0 ⇔ 6

5 ⇔ 4

The transformation from Genome A to Genome B has

4 breakpoints and 2 cycles:

$b = 4 \quad c = 2 \quad \Rightarrow b - c = 2$ ⇔ *2 DCJ*

In (a) caps (shown in square boxes) are placed at the ends of chromosomes for telomeric gene ends. A-caps label telomeric gene ends in genome A, and B-caps label telomeric gene ends in B. In (b) A-B paths have their caps identified, ie 0 is identified with 6, and 5 is identified with 4, and the paths are closed. (c) shows the diagram with uniform colors.

Note that the canonical DCJ capping scheme we arrived at in (c) is different than a naïve capping scheme we might have chosen such as shown in (d). This latter scheme would result in a DIFFERENT DCJ distance since it has $b = 4 \quad c = 1 \quad \Rightarrow b - c = 4 - 1$

⇔ *3 DCJ operations*

Example from Figure 8

Genome A	Genome B
-1, -3, 2	1, 2, 3

(a)

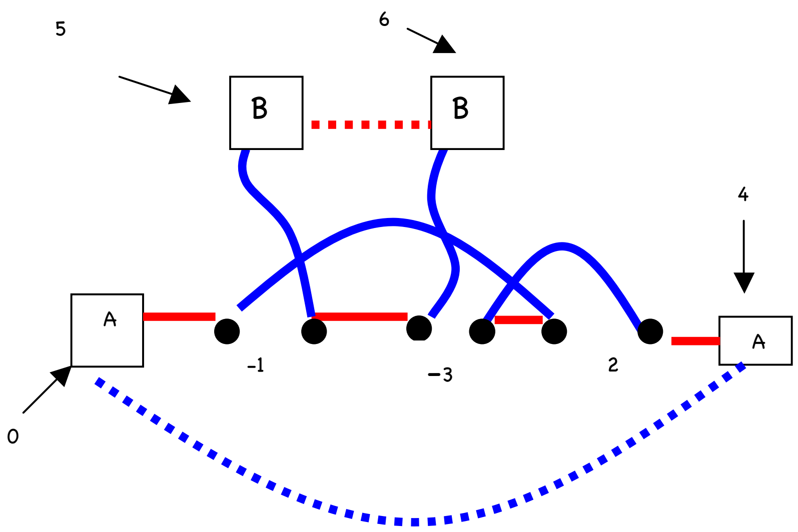

After capping, the genomes become:

Genome A	Genome B
0, -1, -3, 2, 4	5, 1, 2, 3, 6

+ Null chromosomes:

5 6   0 4

(b)

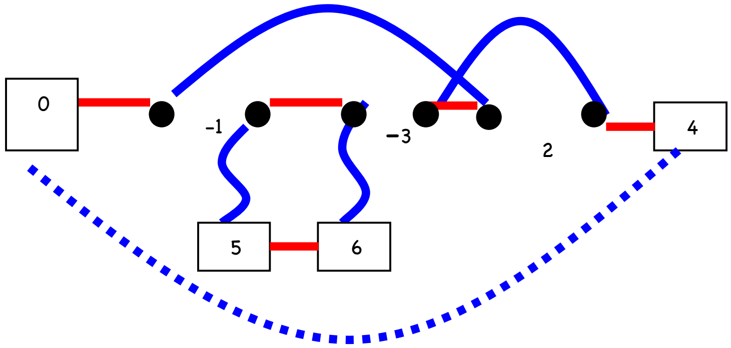

The transformation from Genome A to Genome B has 5 breakpoints and 2 cycles: $b = 5$ $c = 2$ => $b - c = 3$

⇔ *3 DCJ operations*

In (a) we cap genome A with (0) and (4), and genome B with (5) and (6). There is one AA and one BB path. Each is closed by an extra (dotted) line which amounts to creating a null chromosome in the opposite genome providing an extra breakpoint so that b − c = 5 − 2 = 3. The null chromosome in A is (5,6) and in B, (0,4).

(c)

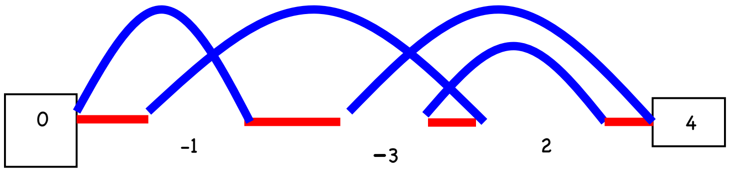

Note that the canonical DCJ capping scheme we arrived at in (b) is *different* than a capping scheme we might have chosen such as shown in (c). This latter scheme would also result in the same DCJ distance since it has

$b = 4$ $c = 1$ => $b - c = 4 - 1$

⇔ *3 DCJ operations*
